# Blind supratemporal retrobulbar block in cats: a feasibility cadaveric study and its efficacy in a group of subjects undergoing corneal or intraocular surgery

**DOI:** 10.3389/fvets.2024.1478732

**Published:** 2024-11-22

**Authors:** Elena Lardone, Manuela Crasta, Pier Carlo Ostan, Paola Gherlinzoni, Alessandra Landi, Paolo Franci

**Affiliations:** ^1^Department of Veterinary Science, University of Turin, Turin, Italy; ^2^AniCura VisionVet, San Giovanni in Persiceto, Italy

**Keywords:** supratemporal, retrobulbar block, cat, corneal surgery, intraocular surgery

## Abstract

**Introduction:**

The supratemporal retrobulbar block (RB) has not been comprehensively described in cats.

**Materials and methods:**

*Cadaveric study*: a modified supratemporal retrobulbar injection of 0.1 ml/kg of iomeprole and saline (1:3) was executed using a Tuohy needle in recently deceased cats. Cadavers underwent computed tomography before and following injections. Injectate distribution within the intraconal space was evaluated. Extraconal injections were considered a failure. *Clinical study*: cats undergoing corneal/intraocular surgery were included. After intramuscular premedication with methadone 0.3 mg/kg, dexmedetomidine 3 mcg/kg and alfaxalone 2 mg/kg and induction with intravenous (IV) alfaxalone to effect, isoflurane anesthesia was maintained with a target end-expired fraction of 1.1%. Cats were randomly allocated in the retrobulbar group [RG, receiving a modified supratemporal RB with 0.1 ml/kg of a mixture of 2% lidocaine (5.5 ml) and 0.75% ropivacaine (2 ml)] or control group (CG). When heart rate or mean arterial pressure increased above 20% of the pre-incisional values, fentanyl (1 mcg/kg IV) was administered. Alfaxalone (0.5 mg/kg IV) was injected when anesthesia was deemed too light. After a total of 3 interventions regardless the type of drugs (fentanyl/alfaxalone), a constant rate infusion of fentanyl (5 mcg/kg/h IV) was started. Anesthetist interventions, quality of recovery (blindly assessed using a descriptive score scale), postoperative eye rubbing, complications were studied.

**Results:**

In the cadaveric study 8 eyes were included (success rate = 87%). The contrast agent spread was scored “moderate-to-large” or “large” in 85.7% of cases and a median 360° (180–360) distribution around the optic nerve was reported. In the clinical study 12 cats were included (6 in RG, 6 in CG). The median time to perform the RB was 35 s (20–50). Only the controls required anesthetist interventions [total amount of 6 (*p* = 0.0276): fentanyl (3/6) and alfaxalone (2/6)]. The RG had a significantly better recovery score (*p* = 0.0012) than CG. Only controls showed eye rubbing (3/6).

**Conclusions:**

The modified supratemporal RB is an achievable and rapidly performed technique. In this study it reduced intraoperative drug administration and improved recovery quality in cats undergoing corneal or intraocular surgery.

## 1 Introduction

The retrobulbar block (RB) consists of deposition of a local anesthetic within the ocular cone to produce analgesia, immobilization, and protrusion of the eye in corneal and intraocular surgeries (1). This locoregional anesthesia technique has been described in several domestic animals (2–4). In cats, it was first described in a cadaveric study (5) using the dorso-medial approach with a manually bent spinal needle, and in six sedated experimental cats the year after (6), showing a lower success rate than using ultrasound guidance to aid the correct needle placement. These studies were based on the study published by Accola (2) in dogs showing that the inferior-temporal palpebral approach was easy to perform and provided complete intraconal coverage without complications. Nevertheless, frontal approaches seem more inspired by the RB technique in humans rather than by the small animal orbital anatomy. In the former, the ocular cone can be easily reached only inserting the needle frontally (7), while in small animals a laterally open orbital cavity offers easy access for puncturing an oblong cranial-caudal directed ocular cone. Unlike the frontal techniques, the lateral approach described by Chiavaccini et al. (8) may have the advantage of positioning the needle well far from the eye bulb, reducing the risk of accidental puncture of the ocular bulb. Additionally, the configuration of the orbital cone, including its length and lateral surface, allows for accurate targeting. Although an ultrasound (US)-guided supratemporal technique has been recently mentioned in a cat complicated with suspected brainstem anesthesia (9), this technique has never been comprehensively described in this species. Some differences in the needle positioning could be caused by anatomical dissimilarity between dogs and cats, such as a more voluminous ocular globe with the respect of the orbital cavity dimensions in the latter.

In clinical practice, the RB is frequently executed in animals undergoing enucleation, however, its area of nerve blockade does not include areas involved in this surgical procedure such as the palpebral tissue, therefore its analgesic activity is inadequate (10). To date there is a lack of clinical studies which evaluate the advantages and disadvantages of performing RB block in cats undergoing ocular surgeries where analgesia can be properly guaranteed by RB such as corneal and intraocular surgeries.

Therefore, the goals of this study were: (1) to evaluate the feasibility and injectate distribution of the supratemporal RB in cat cadavers; (2) to clinically characterize the RB block in in cats undergoing corneal and intraocular surgeries. To do this, we recorded intraoperative anesthetic sparing effect, procedural failure, quality of recovery from anesthesia, and block-related complications.

## 2 Materials and methods

### 2.1 Feasibility and spread evaluation study on blind supratemporal retrobulbar block in cat cadavers

Refrigerated feline cadavers donated to the University of Turin and euthanized/dead for reasons unrelated to this study were included. Excluding criteria were a prolonged refrigeration (>48 h), suboptimal preservation process, and gross ocular, periocular, or orbital abnormalities. Cats not immediately refrigerated after death were also excluded.

All cadavers were positioned in lateral recumbency and the supratemporal area was clipped. All injections were performed by an experienced veterinary anesthesiologist (PF). A modified supratemporal approach was used. A 22G × 50 mm Tuohy needle (Perican; BBraun, Italy) was inserted through the skin between the posterior aspect of the orbital ligament and the zygomatic process of the temporal bone, on the caudo-lateral orbital margin. The needle was advanced medially and slightly ventrally relative to the sagittal plane of the head, with a slight volar direction.

This decision was reached following a preliminary trial in which the rostral needle inclination resulted in bilateral ocular perforation in a cat cadaver.

In this modified technique, the correct positioning of the needle was determined through the observation of a slight rotation of the ocular bulb, produced by the rounded tip of the Tuohy needle pushing against the bulbar cone. This phenomenon was identified as the “rotation sign”. Afterwards, the needle was advanced, the cone was penetrated, and a loss of resistance was perceived by the operator. Subsequently, a repositioning of the bulb was observed. Correct positioning of the needle was confirmed by a computed tomography (CT) scan and the presence of the “rotation sign” was recorded.

A 1:3 mixture of iomeprole (Iomeron 300; Bracco, Italy) and 0.9% sodium chloride solution (Sodio Cloruro 0.9%; Galenica Senese, Italy) was slowly injected at 0.1 ml/Kg.

Three minutes after injection the cat underwent CT imaging to establish the distribution of the contrast agent. CT acquisitions were performed using a Siemens Healthcare Somatom Emotion 16 scanner with following parameters: 1 mm slice, 110 KV, 130 mA, 0.8 Spiral Pitch, Soft and Bone tissue reconstruction algorithms.

Subsequently, the cadaver was repositioned in contralateral recumbency, and the procedure was repeated for the contralateral eye.

A radiologist evaluated the CT images scoring the intraconal volume of distribution according to a modified scale (5) in 5 points (poor = 0; poor/moderate = 0–1; moderate = 1; 1–2 = moderate/large; 2 = large; Table 1). In contrast with the original classification, a five-point scale was selected to delineate the distribution more accurately. The extension of the spread of the injectate around the optic nerve was also graded in 5 points (0, 90, 180, 270, and 360°) as well as the distance between the edges of the contrast medium and the optic foramen was evaluated. The depth of needle penetration was determined by measuring the distance between the animal's skin and the Tuohy needle tip.

**Table 1:** Intraconal spread and distribution around the optic nerve after a modified supratemporal retrobulbar injection of 0.1 ml/kg of iomeprole and saline (1:3) in recently deceased cats. Cadavers underwent computed tomography before and following injections.

**Intraconal spread**	**Description of contrast spread in retrobulbar space**	**Score**
0 (poor)	Punctiform or linear traces	0
0–1 (poor/moderate)	<25%	1
1 (moderate)	25–50%	4
1–2 (moderate/large)	50–75%	1
2 (large)	75–100%	0
**Distribution around the optic nerve**		
0°		0
90°		0
180°		1
270°		1
360°		4

An injection was defined as 'successful' if an intraconal injectate was visible on the CT images.

#### 2.1.1 Statistical methods

Not normally distributed data are reported as median and range. Categorical variables are expressed as frequency and percentage. Statistical analysis was performed using MedCalc Software for Windows version 12.5 (MedCalcSoftware, Ltd., Belgium).

### 2.2 Blind supratemporal retrobulbar block in cats undergoing corneal and intraocular surgery

The clinical trial was approved by the Bioethics Committee of the University of Turin (no. 0421477-29/07/2022). The study was performed at Clinica Veterinaria Visionvet Anicura (San Giovanni in Persiceto, Italy) between August 2022 and September 2023. Written informed consent was obtained and signed by all cat owners.

American Society of Anesthesiologists (ASA) physical status I-III cats undergoing intraocular and corneal surgery were included in this study. The exclusion criteria included aggressive behavior, severe heart disease and any form of eye surgery other than corneal or intraocular procedures, such as eyelid surgery. A comprehensive clinical examination, complete blood count and serum biochemistry were conducted on all animals prior to surgery.

The cats were randomly allocated to receive RB (retrobulbar group, RG) or fentanyl (control group, CG) by simple randomization based on a computer-generated randomization sequence (www.randomizer.org).

In both groups the premedication consisted of intramuscular (IM) methadone 0.3 mg/kg (Semfortan; 10 mg/ml, Dechra, Italy), dexmedetomidine 3 mcg/kg (Dexdomitor; 0.5 mg/ml, Orion Pharma, Finland) and alfaxalone 2 mg/kg (Alfaxan 10 mg/ml, Dechra, Italy). Once sedated, an intravenous catheter (Delta Med, Italy) was placed in the cephalic vein and anesthesia was induced with intravenous (IV) alfaxalone to effect. The trachea was intubated and connected to a circle breathing system. Anesthesia was maintained with isoflurane (Isoflo; Zoetis, Italy) with an end-expired fraction (FE'ISO) of 1.1% in a mixture of oxygen and air (inspired fraction O_2_ 0.4%). Lactate Ringer solution was administered IV at 5 ml/kg/h (Lactated Ringer, Fresenius Kabi, Italy) from intubation to completion of surgery. Pressure-controlled mechanical ventilation was set to maintain an end-tidal CO_2_ (ETCO_2_) between 35 and 45 mmHg (Avance S5 Carestation, GE Healthcare, USA). Heart rate (HR), non-invasive blood pressure (NiBP), respiratory rate (RR), FE'ISO, ETCO_2_, and oxygen saturation (SPO_2_) were continuously monitored (GE Datex-Ohmeda Cardiocap/5 Patient Monitor, GE Healthcare). Data were manually entered on an anesthesia record every 5 min for the entire duration of anesthesia. The animals were positioned on the operating table in dorsal recumbency, with the head rotated slightly toward the non-operational side and lifted by a pneumatic pad at the head of the table.

Periocular clipping was performed in all cats to facilitate blinding in the recovery quality assessment.

In the RG an expert operator (PF) punctured the supratemporal region using a 22G × 50 mm Tuohy needle (Perican; BBraun, Italy) to provide a modified supratemporal RB as described in the cadaveric phase of the current study.

A mixture of 5.5 ml of 2% lidocaine (Lidocaina 2%, Ecuphar, Italy) and 2 ml of 0.75% ropivacaine (Naropina 0.75%, AstraZeneca AB, Sweden) was prepared. Subsequently a dose of 0.1 ml/kg (comprising 1.46 mg/kg of lidocaine and 0.2 mg/kg of ropivacaine) was slowly injected. An attempt to perform the block was defined as repositioning of the needle after its complete extraction. After three consecutive attempts the technique was considered failed, and no further attempts were made.

Cisatracurium (Cisatracurio 0.2%/0.2 mg/kg IV; Mylan Pharma, Italy) was employed as an intraoperative neuromuscular blocking agent when required by the surgeon to improve surgical conditions (i.e., a central and protruded eye). The neuromuscular block was monitored with train-of-four (TOF) stimulation and acceleromyography (AMG), employing a calibrated TOF-AMG monitor (Stimpod NMS 450X, Xavant Technology, South Africa) on one of the hindlimbs. When deemed necessary, neuromuscular block was antagonized with neostigmine (Prostigmina, 0.5 mg/mL, Meda Pharma, Italy; 0.04 mg/kg IV) preceded by atropine (Atropina Solfato, 1 mg/mL, ATI, Italy; 0.02 mg/kg IV). Time needed to perform the block, the presence of the rotation sign, the number of attempts to perform the block, the presence of the eye centralization, and the neuromuscular blocking agent dosing were reported.

Complications related to the locoregional technique were also recorded as well as bradycardia [heart rate (HR) <90 bpm, beats per minute], hypotension [defined as 2 or more consecutive blood pressure readings at an interval of 5 min where mean arterial pressure (MAP) was <65 mmHg], and atropine requirements (20 mcg/kg IV, when HR <90 bpm).

A bolus of fentanyl 1 mcg/kg IV (Fentadon; 50 mcg/ml, Dechra, Italy) was administered as intraoperative rescue analgesia (iRA) if the HR and/or the MAP (for two consecutive measurements corresponding to 5 min) increased by ≥20% of pre-incisional levels. Alfaxalone at the dose of 0.5 mg/kg was administered IV when surgical stimulation produced lighting of anesthetic depth, and the anesthetist perceived an increased risk of unintentional movement by the patient. A continuous infusion of fentanyl at 5 mcg/kg/h was started when the total dose of fentanyl and alfaxalone had reached 3 boluses.

The start and the end of the surgery, the number of boluses of fentanyl and alfaxalone administered, the number of anesthetist interventions, time to extubation (time elapsed from the end of isoflurane administration to extubation), time in sternal recumbency were recorded.

Quality of recovery was evaluated by a trained operator (GP) blind to treatment using a score scale (Annex 1), as described by Jiménez et al. (11). Explanatory comments on recovery could be reported on the recovery sheet by the anesthetists.

#### 2.2.1 Statistical methods

Based on the assumption that the cats receiving RB would require fewer anesthetist interventions than controls, we calculated an effect size of 1 (as suggested by a pilot study), with 6 cats per group to identify a difference in the number of interventions with 95% power and 5% alpha (ClinCalc.com). Categorical variables are expressed as frequency and percentage; Fisher's exact test was used to evaluate frequency distribution independence between the two groups. Not normally distributed data are reported as median and range and were analyzed using the Mann–Whitney U test. Statistical analysis was performed using MedCalc Software for Windows version 12.5 (MedCalcSoftware, Ltd., Belgium). Statistical significance was set at 5%.

## 3 Results

### 3.1 Feasibility and spread evaluation study on blind supratemporal retrobulbar block in cat cadavers

Four adult cat cadavers were included: 1 female and 3 males weighting 4.2 kg (3.7–7.8).

One eye was excluded because a peribulbar diffusion of the injectate was observed. This was arranged around the eyeball in a manner akin to a crown, clearly defining its margins (success rate 7/8, 87%).

Intraconal spread and distribution around the optic nerve are shown in Table 1.

Based upon imaging data, in one eye it was not possible to ascertain the distribution around the optic nerve. Median contact area around the optic nerve was 360° (180°-360°) and median depth of needle penetration was 2.5 cm (2.5–2.6) (Supplementary Figure 1). The median distance between the edges of the contrast medium diffusion and the optic foramen was 3 mm (3–8). Regarding the rotation sign, it was evident in three cases, undetected in one, and barely perceptible in the remaining three.

In one eye, the spread of the contrast medium in a lateral position was observed, which led to the suspicion of its exit from the muscular cone. Additionally, in the same animal, a particular proximity of the caudal margin of the contrast medium to the optic foramen was also observed.

### 3.2 Blind supratemporal retrobulbar block in cats undergoing corneal and intraocular surgery

Twelve privately-owned cats were enrolled in the study and allocated to the two groups (6 in RG and 6 in CG). No cats was excluded. Patient demographics and surgical procedures are showed in Tables 2, 3, respectively.

**Table 2:** Patient demographics in the retrobulbar group [RG, receiving a modified supratemporal retrobulbar block with 0.1 ml/kg of a mixture of 2% lidocaine (5.5 ml) and 0.75% ropivacaine (2 ml)] and control group (CG, no block)—DSH, domestic shorthair; ASA (American Society of Anesthesiologists) physical status classification.

**Characteristic**	**RG (n 6)**	**CG (n 6)**	**Total**	***p*-value**
**Breed (** ***n*** **)**				
DSH	4	3	7	
Persian cat	1	2	3	
Exotic shorthair	1	1	2	
Age (years)	9 (1–15)	10 (1.5–14)		1
Weight (kg)	5 (3.5–5.3)	5 (2.7–5.7)		0.289
**ASA Class**				
I	5	4	9	1
II	1	2	3	

**Table 3:** Surgical procedures in the retrobulbar group [RG, receiving a modified supratemporal retrobulbar block with 0.1 ml/kg of a mixture of 2% lidocaine (5.5 ml) and 0.75% ropivacaine (2 ml)] and control group (CG, no block)—ISP, intraocular silicon prosthesis.

**Surgery**	**RG (n 6)**	**CG (n 6)**	**Total**	***p*-value**
Corneal (*n*)	5	3	8	0.545
Keratectomy	3	1	4	
Corneal perforation	0	1	1	
Corneal reconstruction	2	1	3	
Intraocular (*n*)	1	3	4	0.545
Corneal sequestration	1	1	2	
ISP	0	1	1	
Iris biopsy	0	1	1	

The RB was performed in a median time of 35 s (range 20–50) and required a single attempt. The rotation sign was detected in 4/6 cats and eye centralization was present in all RG cats. No failure neither complication was observed. No differences were detected between groups regarding procedural data (Table 4).

**Table 4:** Procedural data in the retrobulbar group [RG, receiving a modified supratemporal retrobulbar block with 0.1 ml/kg of a mixture of 2% lidocaine (5.5 ml) and 0.75% ropivacaine (2 ml)] and control group (CG, no block).

**Characteristic**	**RG (n 6)**	**CG (n 6)**	***p*-value**
Median time for RB (sec) (range)	35 (20–50)	NA	NA
Attempts (*n*)	1 (1)	NA	NA
Failures (*n*) (%)	0/6	NA	NA
Duration of anesthesia (min) (range)	64 (23–167)	67 (29–169)	0.818
Duration of surgery (min) (range)	20 (5–68)	31 (8–60)	0.809
Extubation time (min) (range)	6 (2–21)	8.5 (1–34)	0.719
Sternal Recumbency (min) (range)	10.5 (7–27)	8 (5–17)	0.926

The CG required a median anesthetist intervention of 1 (0–2) whereas no such interventions were necessary in RG, resulting in a statistically significant difference between groups (*p* = 0.0276).

The median recovery score was statistically lower (i.e., better) in the RG than in the CG (*p* = 0.0012): 2 (1–2) in RG and 3 (2–3) in CG. During anesthesia recovery, attempts to rub eyes were noted in 3/6 control cats and none in RG (*p* = 0.1812) (Table 5).

**Table 5:** Intraoperative rescue analgesia (iRA) and Recovery score in the retrobulbar group [RG, receiving a modified supratemporal retrobulbar block with 0.1 ml/kg of a mixture of 2% lidocaine (5.5 ml) and 0.75% ropivacaine (2 ml)] and control group (CG, no block).

**Characteristic**	**RG (n 6)**	**CG (n 6)**	***p*-value**
iRA (*n*) (%)	0 (0%)	3 (50%)	NA
Median fentanyl dose (mcg/kg) (*n*) (range)	0	1 (0–3)	0.073
Median time to first fentanyl bolus (min) (range)	0	14 (5–19)	NA
Alfaxalone (*n*) (%)	0	2 (33.3%)	NA
Median alfaxalone (mg/kg) (range)	0	0 (0–1)	0.173
Median time to first alfaxalone bolus (min) (range)	0	10 (5–15)	
Total anaesthetist's interventions (*n*)	0	6	NA
Median anaesthetist's interventions (*n*) (range)	0	1 (0–2)	**0.0276**
Cisatracurium (*n*)	0	0	NA
Median recovery score	2 (2)	3 (3)	**0.0012**
Eye rubbing (*n*) (%)	0	3 (50%)	0.181

Bold values are statistically significant (*p* < 0.05).

Neither intraoperative complications nor atropine usage was recorded. The median HR was not different between groups [104 (94–142) in RG and 109 (84–150) in CG], whereas the median MAP was higher in CG [83 (62–116) in CG and 68 (62–79) in RG; *p* = 0.748] although this difference was not statistically significant (*p* = 0.198).

The RG cats did not receive iRA (0/6) whereas half of the controls required it (3/6): the median fentanyl consumption (mcg/kg) was 0 in RG and 1 (0–3) in CG, but this difference was not statistically significant (*p* = 0.0731).

Although alfaxalone was administered only in CG (2/6) with a median dose of 0 mg/kg (0–1), no statistical difference was detected (*p* = 0.1739) between groups.

## 4 Discussion

This clinical study is one of a limited number of studies that investigate the use of a RB in cats undergoing corneal and intraocular surgeries. The main findings of this study are that the modified supratemporal RB technique in cats is feasible and effective not only in reducing the intraoperative anesthetist's interventions, but also in improving the quality of anesthesia recovery.

The anesthesia was maintained throughout the surgical procedure with a targeted FE'Iso of 1.1% in both groups. The more stable anesthesia in the RG (no iRA and no alfaxalone administrations) meant that the block represents a superior compromise between depth of anesthesia (with consequent cardiovascular depression) and nociceptive stimulation control. Furthermore, this result is particularly interesting in ocular surgery where anesthesia lightning and patient movements may have a detrimental impact on the surgical outcome.

The efficacy of regional analgesia in facilitating a better recovery from anesthesia has been widely documented (12–15). In the context of ocular postsurgical patients, where there is a potential risk of self-harm negatively impacting surgical outcomes, regional techniques may be particularly advantageous. Furthermore, there is evidence to suggest that these techniques may reduce the risk of poor recovery and subsequent damage to the operated eye (16, 17). In this study, none of the RG cats tried to rub its eyes during recovery. The intense and sufficiently prolonged analgesia produced by the RB may have exerted a good level of anesthetic recovery, which reduced the risk of self-harm. We believe that this is probably the main benefit to perform RB in animals undergoing corneal and intraocular surgeries, as suggested for pediatric patients, other non-collaborative subjects, where RB was found a better alternative to systemic fentanyl (18, 19). Furthermore, a more straightforward recovery from anesthesia may contribute to a safer and potentially more efficient work in the operating room.

We believe that one factor limiting the use of RB in veterinary ocular surgery is the risk of complications, such as chemosis, ecchymosis, retrobulbar hematoma, increased intraocular pressure (IOP), central spread of local anesthetic, brain stem anesthesia, ocular globe perforation, and optic nerve damage.

According to human literature such risks are quite scarce: the number of life-threatening events after regional anesthesia techniques for eye surgery is low at 3.4 per 10,000 cases in humans (20) and the incidence of minor complications (e.g., retrobulbar hematoma) is 0.04% to 1.7% (21, 22). In the current study, data on the small-sized RG do not allow for speculation about the technique's real safety. While supratemporal RB may have a lower incidence of ocular bulb perforation (due to the farther needle positioning from the ocular globe and the rotation sign, as self-control of the technique), the deposition of local anesthetic close to the optic foramen could theoretically increase the risk of brainstem anesthesia. In the cadaveric study, the distance between the margins of the contrast spread and the optic foramen was found to be a notably short (3 mm). A case of brainstem anesthesia was, in fact, reported after US-guided RB via the supratemporal approach in a cat (9). The rounded shaped tip of the Tuohy needle should be able to lower the risk of damaging vessels (23) and nerves within the cone, as hematoma is one of the most common complication due to RB technique. Furthermore, the 22G Tuohy is an optimal balance between the safety of the rounded tip and a moderate degree of sharpness due to the small caliber, which enables effortless penetration of the cat's hard skin.

In the pilot study, bilateral perforation of the ocular globe was observed in the first cadaver because the needle direction was aligned with the approach described by Chiavaccini et al. (8) in the dog. In comparison with the dog, the anatomy of the feline eye is characterized by a more voluminous ocular globe with the respect of the orbital cavity dimensions. To prevent further accidental punctures of the globe, in our modified technique, we directed the needle slightly more caudally than that described by the aforementioned study. It is noteworthy that the depth of needle penetration was found to be consistently uniform within the sample (2.5 cm). This information could potentially contribute to enhancing the safety of providing this block.

The results of the cadaveric study demonstrated that the modified supratemporal RB in cats was a viable procedure, with a moderate-to-large or large contrast agent spread in the majority of cases (85.7%) and a good distribution around the optic nerve. It is very likely that the short time taken for performing the block (around 35 s on average) and the high success rate are mainly due to the lateral approach which allows an easy puncture of the ocular cone, the presence of the loss of resistance and the rotation sign when present. From this perspective, the use of a Tuohy needle is of paramount importance, as the rounded tip transmits a sensation of the consistency of different tissue layers, particularly the ocular cone. The operator, upon sensing that the needle tip is in contact with the cone, may have greater certainty whether performing peribulbar or retrobulbar injection.

Ultrasound imaging is a valuable tool in locoregional anesthesia, as it allows for precise control of needle tip positioning and accurate deposition of local anesthetics. Based on the literature search, few cadaveric studies (24, 25) and clinical studies have been published on US-guided RB block in dogs (26, 27) and cats (28). Given that the spread of local anesthetic is predictable due to its injection into an anatomical space delineated by connective fascia and that our modified technique is guided by ocular movements in relation to needle advancement, the RB as performed here appears to be a reasonable approach for providing a locoregional block in a body area where the use of a US-guided technique is not always straightforward.

A solution of lidocaine and ropivacaine was employed, with a final concentration of 2 mg/ml ropivacaine. This decision was made to achieve a balance between two important considerations: the need for a rapid onset of action and the avoidance of potential complications associated with maintaining an awake cat with an immobile, central, protruded, and insensitive eye. In a study conducted by Chin (29), the mean time for eye akinesia in humans undergoing RB with lidocaine 2% (3 ml), epinephrine, and hyaluronidase was reported to be 4 h. A low concentration of ropivacaine was administered with the objective of prolonging the analgesic effect observed in the postoperative period. The practice of combining local anesthetics is a topic of debate in both human and veterinary medicine. Although combinations of local anesthetics have been employed in clinical practice for a considerable period of time in humans, the rationale for combining the drugs remains unclear, particularly in the context of epidural and peripheral nerve blocks. It is inaccurate to assume that the result of combining two distinct local anesthetics to perform a nerve block is consistent across different locoregional techniques. Several factors including anatomy, tissue perfusion, the total amount of drug administered, and the type of nerve can influence the onset, duration, and intensity of a block. The effects of a local anesthetic injected into the retrobulbar space or into the brachial plexus can differ. In order to provide a RB, the local anesthetic is injected into the ocular cone, which is a physically delimited anatomical space where the thin nerves are contained in a connective fascia and therefore readily and completely bathed by the drug. This situation bears similarity to that of the subarachnoid space, wherein the spinal cord is enclosed within the arachnoid meninges. In humans undergoing RB or spinal block, the addition of lidocaine to bupivacaine yielded different outcomes compared to the administration of a single drug. In RB, the onset was faster and the duration longer. In spinal block, the onset was similar, but the duration of the block was longer (30, 31). A review of the literature revealed no studies employing the identical local anesthetic mixture. As the postoperative effects of the mixture were not evaluated in this study, it is not possible to ascertain the value of adding ropivacaine.

Regarding the potential for unpredictable toxicity of a mixture of local anesthetics, the dose of ropivacaine (0.2 mg/kg) employed was insufficient to give cause for concern. Furthermore, the pH of the lidocaine-ropivacaine combination is similar, which allows for the reasonable assumption that its pharmacokinetics may not differ considerably.

It was decided that ml/kg would be used in preference to mg/kg doses, to ensure simplicity. A combination of two drugs would have been unduly lengthy and complex to report the doses for each local anesthetic. Nevertheless, this approach may prove beneficial in routine clinical practice. The mixture, comprising 0.2 mg/kg of ropivacaine and 1.46 mg/kg of lidocaine, was administered at a dose of 0.1 ml/kg.

Previously studies reported a larger volume of anesthetic (total dose: 1 ml) (5, 6). Our choice on the anesthetic volume (0.1 ml/kg) was based on the compromise between a good anesthetic spread and a limited increase in intraocular pressure (IOP) and risk for brainstem anesthesia. Although a favorable intraconal distribution of contrast medium could be achieved with this volume, as demonstrated by the CT images obtained during the cadaveric study, further studies are needed to investigate the optimal injectable volume. It is important to understand that although the study of contrast agent distribution in cadavers could provide a rough indication of the distribution on the local anesthetic solution, *in vivo* spread and nerve block results may be different to in cadavers.

This study has some limitations.

Corneal lesions requiring surgical intervention may be attributed to a range of clinical scenarios, depending on the severity of pain and inflammation. It should be noted that the present sample was not stratified for such events, which may have introduced a potential source of error.

Intraocular pressure was not measured. It is reasonable to hypothesize that the introduction of a quantity of local anesthetic into the orbital cavity would be expected to result in an observable elevation in IOP. Indeed, a rise in IOP has been demonstrated following peribulbar anesthesia in humans (32). Nevertheless, a reduction in IOP has been demonstrated following sub-Tenon blocks, potentially due to a decrease in muscle tone (33). The use of sedation and general anesthesia in veterinary medicine presents a challenge when attempting to study potential changes in IOP. Hofmeister et al. (34) reported that the ventromedial rotation of the eye caused by propofol induction resulted in inaccurate IOP readings in most of the dogs. In a study conducted by Hazra (35), no changes in IOP were observed in dogs anesthetized with xylazine, ketamine and diazepam following RB anesthesia. This was noted at both 6 h and 24 h post-surgery.

The anesthesia protocol used in both groups (light premedication and fixed FE'ISO) provided a light antinociceptive plane to better evaluate the analgesia provided by the RB. On the other hand, this choice may have increased the risk of confounding nociceptive response with inadequate hypnosis.

This study aimed to assess intraoperative nociception (not conducted with blinding) and anesthetic recovery (conducted by a blinded investigator). Further research should be conducted to gain a greater understanding of the extent to which the RB provides postoperative analgesia.

## 5 Conclusions

The modified supratemporal RB in cats undergoing ocular surgery was found to be a rapidly performed technique providing a more stable anesthesia and a favorable recovery profile.

## Data Availability

The original contributions presented in the study are included in the article/Supplementary material, further inquiries can be directed to the corresponding author.
