# Supplementary figures and images for: Blind supratemporal retrobulbar block in cats: a feasibility cadaveric study and its efficacy in a group of subjects undergoing corneal or intraocular surgery
Source: Front Vet Sci. 2024 Nov 22;11:1478732. doi: 10.3389/fvets.2024.1478732 (PMC11621063; doi:10.3389/fvets.2024.1478732)

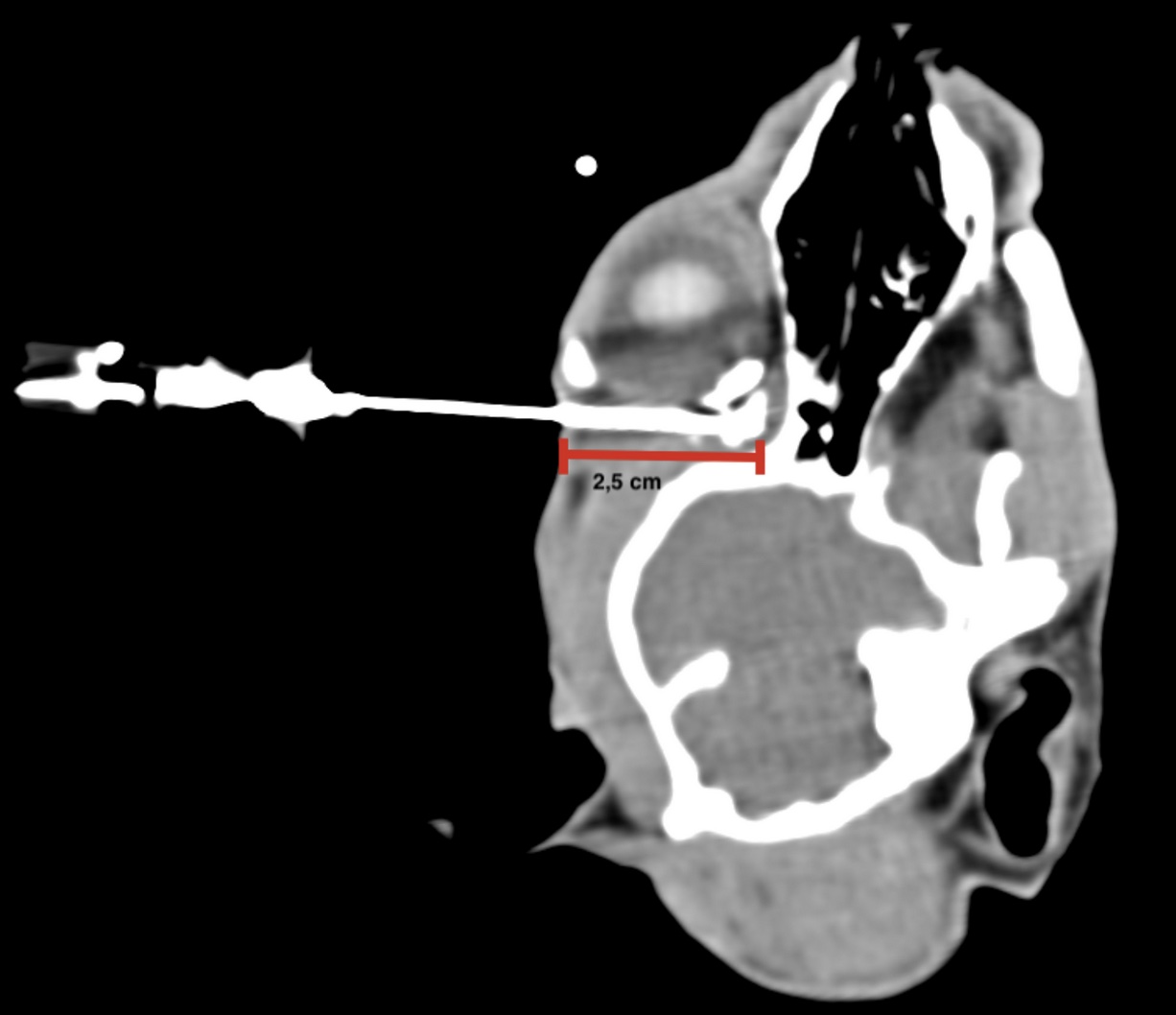

Supplement: Supplementary file 1 [file Image_1.JPEG]
